# Synthetic learning machines

**DOI:** 10.1186/s13040-014-0028-y

**Published:** 2014-12-18

**Authors:** Hemant Ishwaran, James D Malley

**Affiliations:** Division of Biostatistics, University of Miami, 1120 NW 14th Street, Miami, 33136 FL USA; Center for Information Technology, National Institutes of Health, Bethesda, 20892 MD USA

**Keywords:** Machine, Nodesize, Random forest, Trees, Synthetic feature

## Abstract

**Background:**

Using a collection of different terminal nodesize constructed random forests, each generating a synthetic feature, a synthetic random forest is defined as a kind of hyperforest, calculated using the new input synthetic features, along with the original features.

**Results:**

Using a large collection of regression and multiclass datasets we show that synthetic random forests outperforms both conventional random forests and the optimized forest from the regresssion portfolio.

**Conclusions:**

Synthetic forests removes the need for tuning random forests with no additional effort on the part of the researcher. Importantly, the synthetic forest does this with evidently no loss in prediction compared to a well-optimized single random forest.

## Background

Earlier work has shown how to optimally combine a set of predictors—classifier or probability machines—into a so-called *regression collective* [1]. Consider, for example, a collection of statistical learning machines chosen by the researcher, from subject matter knowledge or with statistical aspirations. It could contain versions of SVMs [2] with different kernels, variants of the lasso [3], a group of neural nets [4], a collection of *k*-nearest neighbors [5] with varying *k*, along with several random forests [6]. The thought here is that each or several of these machines might be optimal for the data at hand, but tuning each and adjudicating the multiple outcomes and performances introduces a second layer of statistical and data analytic overhead. Instead, a prefered way to proceed is to use a regression collective which optimally combines machines, thus avoiding the difficulty of individual machine tuning.

This new method [1], whose R code is abbreviated COBRA (for COmBined Regression Alternative), is just this kind of combining method. It has the property, that in the limit of large data, it is at least as good as the best predictor in the collection, and generates its prediction without having to declare which of the individual predictors might be optimal for the data at hand. For example, on a given data set one method might be Bayes optimal for classification given enough data, and on another data set another machine might be optimal. In all cases the regression collective, given enough data, is Bayes optimal if any one machine is so, and where this unnamed optimal machine in the portfolio of the collective can vary over data sets. The method achieves this optimality for any data, with a possibly large number of features with mixed category or continuous features and arbitrary correlation structure. As a practical matter the regression collective requires no tuning of the individual machines on the part of the researcher: the method is entirely nonparametric and model-free. As one detail, there is no requirement for specific and correct interaction terms, however defined, as input for the method. Instead, machines that include such interaction terms can be added to the portfolio of the collective.

The COBRA method is not a committee or ensemble method, nor is it a voting method. It is closer to a *k*-nearest neighbor scheme. However the distance function, or metric, for measuring closeness in the collective is not Euclidean distance or any weighting thereof, but a method that uses the multiple predictions of the several component machines to access closeness of a test data point to the training data. For each training data point, one checks if its predicted value under a given machine is close to the predicted value of the test data point under that same machine, and if closeness holds for a majority of the machines, then that training point is deemed close to the target test data point, otherwise it is deemed distant. The final prediction for the test data point is a sum of the training data outcomes using only those data points that are close. In particular this means predictions from the several machines are not averaged to make the prediction on the test case, but rather the predicted value is a weighted average of the original outcomes. That is, COBRA is a type of locally weighted averaged estimator.

While the example above of a regression collective over a set of learning machines was the motivation for COBRA [1], it has also lead to increased scrutiny of analytic approaches using collections of features, biologically grounded networks or pathways each as new inputs to other machines. Here the separate networks are used as inputs to the various machines within the collective [7]. Then, using the machines built from these networks as *synthetic features*, they can be sent to a suitable learning machine for which it is then possible to compare and evaluate the predictive capacity and interactions between the networks: Are some networks better than others in the portfolio? In what subset of the data might that be true?

The approach described here links these two methods, that of a regression collective and the introduction of synthetic features. We describe a *synthetic machine approach*, in particular an approach we call *synthetic random forests*. Using a collection of differently tuned random forests, each generating a synthetic feature, a synthetic random forest is defined as a secondary random forest, a kind of hyperforest, calculated using the new input synthetic features, along with all the original features. The motivation for using random forests as a combiner is motivated by the COBRA approach. Like COBRA, a random forest can be described as a locally weighted averaged estimator, however it differs from COBRA in that the weights used to average training outcomes are arbitrary convex weights, whereas COBRA weights are either zero or one values. It is the greater flexibility afforded by convex weights that is the rationale for considering random forests as a combiner. We study the properties of this new synthetic forest method using large scale simulations involving both real and synthetic data. We find the method has the similar property to COBRA that it appears to be as universally as good, across all our test data sets, as the optimal machine in the portfolio of its collective. But not only that, our empirical findings also suggest that the synthetic random forest outperforms the orginal COBRA regression collective scheme.

## Methods

Random Forests [6] (hereafter abbreviated as RF), is an ensemble learning method which calculates ensemble predicted value by aggregating a collection of *ntree*≥1 randomly grown trees. In multiclass problems, averaging the terminal node relative frequency of class labels over a forest of random classification trees yields ensemble predicted probabilities for each class label, while in regression problems, averaging the terminal node mean value yields ensemble predicted values for the *Y*-response. Equivalently, one can show that the resulting ensemble predicted value of a RF can be written as weighted convex combination of outcomes. Thus, RF is a locally weighted averaging estimator. A unique feature of RF trees are that they are random in the following sense: a) Each tree is grown using an independent bootstrap sample (i.e. a sample drawn with replacement from the original data set, of size *n* equal to the original sample size); (b) Random feature selection is employed in which at each node of the tree during the tree growing process, a random subset of 1≤*mtry*≤*p* features are selected, where *p* equals the total number of features, and the node is split using the variable from the *mtry* candidate variables having the best split. Splitting of a RF tree is repeated recursively, with the tree grown as far as possible until it is no longer possible to identify groups that differ on the outcome, or the sample size at that node is too small. Terminal node sizes (the ends of the tree) satisfy the condition that they contain a minimum of *nodesize*≥1 unique cases.

Of the three tuning parameters used by RF, (*ntree*,*mtry*,*nodesize*), optimal tuning of *nodesize* has the greatest potential to improve prediction performance. This is because *nodesize* acts as a type of bandwidth parameter that controls the level of smoothing of the RF predictor. It has now become apparent to the machine learning community that the optimal choice for *nodesize* depends heavily on the underyling data. In large *n* sample settings for example, it is generally believed that *nodesize* should be large to ensure good performance. Rationale for this comes from large sample asymptotics which require *nodesize* to increase to *∞* in order to ensure consistency. Results of this nature have for example been used to establish Bayes-risk consistency for RF classification [8]. On the other hand, in high-dimensional problems involving a large number of features, the opposite has been observed, with performance generally improving with decreasing *nodesize* [9]. In studying lower bounds for the rate of convergence in RF regression, it has been shown that rates of convergence improve when *nodesize* is small when the number of features *p* exceeds the sample size *n* [10].

It is not hard to imagine settings where the underlying target function *f* of interest has curvature that varies over the feature space. Therefore, given that *nodesize* functions as a type of bandwidth smoothing parameter, it stands to reason that an adaptive *nodesize* value that becomes large or small depending upon the flatness or wiggliness of *f* will yield a RF that has the potential to outperform a conventional forest constructed using a single fixed *nodesize* value. In order to allow RF to achieve this type of local adaptivity, our idea is to create synthetic features, which themselves are constructed from forests calculated using different *nodesize* values. This then allows node splits of a synthetic RF tree to make local and adaptive decisions about *nodesize* by selecting from features constructed from different *nodesize* values. It is this key observation that forms the basis of the synthetic random forest (SRF) method described below in Algorithm 1.



### Remarks

Implementing SRF conveniently involves doing nothing more than fitting a RF to a slightly expanded set of features, which includes in addition to the original *p* features, a new collection of synthetic features obtained by fitting RF under different *nodesize* values. While conceptually straightforward, there are some important points to keep in mind when implementing SRF:

1. The dimension of a synthetic feature can be one or greater. In regression, the synthetic feature is the predicted value of the *Y*-response, which is one-dimensional, however in multiclass problems, the synthetic feature is the predicted probability of the class label. If there are *J* classes, this yields a *J*-dimensional synthetic feature. Note that since the predicted probabilities are linearly dependent as they sum to 1, we discard by convention the last coordinate and use only the first *J*−1 predicted probabilities.
2. To avoid overfitting, when constructing the synthetic feature, we use out-of-bag (OOB) predicted values. In a bootstrap sample only 63.2% of the data is used on average (due to sampling with replacement), leaving 36.7% of the data untouched. This latter data is termed OOB because it is out of sample and can be used to calculate OOB ensemble predicted values. The OOB predicted value for each data point **X** does not use the *Y*-response for **X** and therefore represents a cross-validated out-of-sample estimate.
3. The values of *ntree* and *mtry* are kept fixed throughout. Selecting a reasonable value for *ntree* is not difficult and performance is robust to its choice—as long as its value is kept reasonably large, say 250 or more. Optimizing over *mtry* can improve RF but we have found that creating synthetic features by varying both *mtry* and *nodesize* values can sometimes negatively impact performance of SRF. We find keeping *mtry* fixed at default values and constructing synthetic features by varying *nodesize* works very well.
4. Another reason for favoring optimization of *nodesize* rather than *mtry* is its granularity. For *nodesize*, regardless of *n* or *p*, it suffices to consider a handful of small values, a few intermediate values, and a few large values in the optimization (in the case of large *n*, the rate at which *nodesize* converges to *∞*, required for consistency, can be far slower than *n*; thus relatively small values of *nodesize* can be used even when *n* is relatively large). In contrast, optimization over *mtry* depends upon *p*, which creates not only an expensive optimization problem in high-dimensions, but also the potential for overfitting due to the addition of a large number of synthetic features.

## Results

We compared the performance of four methods, RF, RFopt, SRF, and COBRA over a collection of regression and multiclass benchmark datasets. The four methods were defined as follows:

1. SRF denotes Synthetic Random Forests described in Algorithm 1. Values for *nodesize* were set at . The synthetic RF of line 4 was calculated using *nodesize*=5. While this value can be included as a user parameter for SRF, we found that changing its value did not alter our findings very much. Thus we chose not to cloud our findings and instead opted for a fixed value of *nodesize*=5 throughout our simulations.
2. RF denotes a standard forest calculated using *nodesize*=5. The same *nodesize* value was used as for the synthetic forest in SRF in order to assess the efficacy of the synthetic features. If the synthetic features are not used in node splitting of a synthetic forest, the resulting forest should closely approximate a regular forest, and thus performance of SRF should closely approximate performance of RF.
3. RFopt denotes the forest calculated using the optimal nodesize from . Specifically: the optimal nodesize was defined as the nodesize value *n*_*j*_ from the RF_*j*_ forest with the smallest OOB error in SRF. We include RFopt to assess whether a globally nodesize-optimized forest can compete with the locally nodesize-optimized synthetic forest.
4. COBRA implements the aggregation method described in [1]. For regression machines required as input to COBRA we used the same $\{\text {RF}_{j}\}_{1}^{D}$ machines used by SRF. Using the same synthetic features as SRF allows us to assess the effectiveness of arbitrary convex combination weighting used by RF compared with zero-one weighting used by COBRA. As a side note, we also tried implementing COBRA using the default machines that comes with its code (lasso, ridge regression, SVM and random forests) to assess whether a generic COBRA implementation compared favorably to SRF. However, the results were so unfavorable that we excluded them from our findings.

All forests were calculated using *ntree*=500 and *mtry*=[*p*/3] where [*z*] denotes the first integer greater than *z*. Forest computations were implemented using the R-package randomForestSRC [11] which has been extended to include the function rfsrcSyn which implements the Synthetic Random Forests described in Algorithm 1. We note that while forest calculations could have been implemented using other random forest packages, such as randomForest [12], we prefer to use randomForestSRC as it has many useful features for reducing computational times, such as parallel processing using the OpenMP protocol (which we employed), and non-deterministic random splitting via its nsplit option (however while this option is available in the rfsrcSyn function, it was not used here to avoid clouding the issue of tuning parameters). COBRA was implemented using the R-package COBRA [13]. Calibration of the COBRA *ε*-parameter which is recommended to improve performance was implemented by selecting a grid consisting of 200 points. Note that because the COBRA package has not yet been extended to encompass multiclass problems, the COBRA method was excluded from our multiclass experiment.

### Regression results

A large collection of regression datasets was used to assess the performance of each method (Table 1). Datasets with a capital identify real data while those in lower case are synthetic data. Many of the synthetic data were obtained from the mlbench R-package [14] and are labeled starting with “mlb”. In total, 46 datasets were used with sample sizes varying from *n*=31 to *n*=1114; number of features varied from *p*=2 to *p*=500. Sample sizes for synthetic data were set at *n*=250.

**Table 1:** **Regression benchmark performance**

	***n***	***p***	**COBRA**	**RF**	**RFopt**	**SRF**
Air	111	5	27.24	28.68	27.53	28.14
Air2	111	5	28.40	30.72	28.85	28.36
Automobile	193	29	9.83	8.94	6.79	7.52
Bodyfat	252	13	31.36	32.02	31.67	32.19
BostonHousing	506	13	18.88	14.64	12.39	12.80
BostonHousing2	506	16	17.44	13.57	11.32	11.61
CMB	899	4	96.33	100.90	90.32	89.86
Crime	47	15	61.74	59.99	59.51	59.03
Diabetes	442	10	57.58	53.22	53.14	55.20
DiabetesI	442	64	57.05	54.42	54.61	55.92
Fitness	31	6	83.34	66.48	59.61	57.76
Highway	39	11	38.84	43.67	33.95	32.18
Iowa	33	9	62.60	62.16	50.03	50.22
Ozone	203	12	26.90	26.19	26.20	26.42
OzoneI	203	134	27.42	26.14	26.32	26.08
Pollute	60	15	49.64	51.36	49.52	46.74
Prostate	97	8	87.32	46.02	46.95	50.12
Servo	167	19	15.22	21.47	11.27	11.99
ServoFactor	167	16	43.24	34.65	32.54	31.44
Tecator	215	22	13.84	16.11	13.48	6.19
Tecator2	215	100	31.24	34.21	30.64	27.94
Windmill	1114	12	31.64	31.39	31.31	32.15
expon	250	2	47.76	46.04	46.48	47.60
expon.noise	250	17	62.13	67.49	66.44	53.04
mlb.friedman1	250	10	21.46	26.11	24.15	19.04
mlb.friedman1.noise	250	10	30.91	34.77	33.13	30.48
mlb.friedman1.bigp	250	250	37.67	44.14	43.81	31.99
mlb.friedman2	250	4	13.94	14.75	14.24	14.04
mlb.friedman2.noise	250	4	37.19	36.77	36.80	38.58
mlb.friedman2.bigp	250	254	22.92	29.01	28.10	17.73
mlb.friedman3	250	4	19.21	22.01	19.87	15.59
mlb.friedman3.noise	250	4	37.47	39.38	38.53	36.97
mlb.friedman3.bigp	250	254	37.19	46.72	45.47	26.78
mlb.peak	250	20	14.75	17.24	16.28	6.21
mlb.peak.bigp	250	20	14.75	17.24	16.28	6.21
mlb.noise	250	500	101.69	100.75	100.47	100.29
sine	250	2	35.92	37.79	35.95	34.72
sine.noise	250	5	56.64	66.07	61.14	54.71
syn.ex1	250	50	20.69	30.87	28.57	8.54
syn.ex2	250	20	88.60	89.66	89.59	92.68
syn.ex3	250	50	43.88	47.88	47.50	43.04
syn.ex4	250	50	34.75	37.78	36.90	30.40
syn.ex5	250	20	62.50	65.07	64.82	62.80
syn.ex6	250	30		102.30	100.58	103.16
syn.ex7	250	300	55.13	61.68	61.38	52.41
syn.ex8	250	50	117.93	58.11	57.76	52.01

Cross-validated and test-set standardized mean-squared error (MSE) performance over 100 independent replications. Standardized MSE obtained by dividing MSE by the variance of the *Y*-response and multiplying by 100.

**Figure 1:**
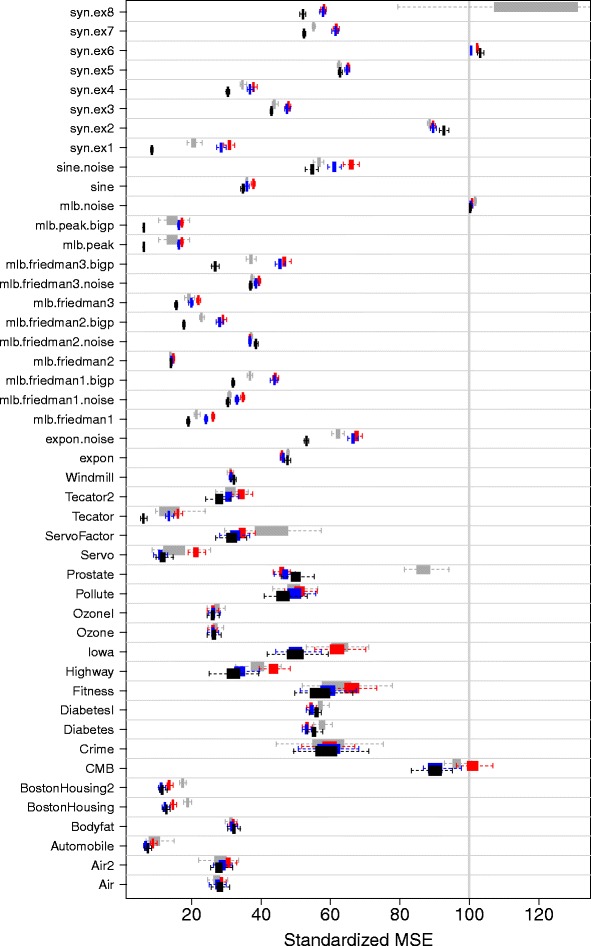
**Regression benchmark results.** Cross-validated and test-set standardized mean-squared error (MSE) performance over 100 independent replications. Boxplots display results from the 100 replications for COBRA (gray square symbol), RF (red square symbol), optimized random forests RFopt (blue square symbol), and synthetic random forests SRF (■). Standardized MSE obtained by dividing MSE by the variance of the *Y*-response and multiplying by 100.

Performance was assessed using standardized mean-squared error (MSE) defined as MSE divided by the variance of the *Y*-response and multiplied by 100. Standarized MSE facilitates comparison across datasets: a value of 100 can be used as a benchmark value. For real data, MSE was calculated using 10-fold cross-validation. For synthetic data, MSE was evaluated by using an independent test-set of size *n*=5000. The entire process was repeated independently 100 times. Table 1 reports the averaged standardized MSE from the 100 replicates. Figure 1 displays the 95% confidence regions of standardized MSE.

Table 1 and Figure 1 show clear superiority of SRF, especially over synthetic data. To formally assess performance differences we used univariate and multivariate nonparametric statistical tests [15]. To compare two methods we used the Wilcoxon signed rank test applied to the difference of their standardized MSE values. The exact p-value for the Wilcoxon signed rank test are recorded along the upper diagonals of Table 2. The lower diagonal values record the corresponding test statistic where small values indicate a difference. To test for an overall difference among procedures we used the Iman and Davenport modified Friedman test [15]. For each dataset, the performance of each method was ranked from 1 through 4, and the average of these ranks over all datasets for each procedure calculated. The diagonal values of the table record this average rank which was used for the Friedman test. This latter test yielded a near-zero p-value, thus providing strong evidence of difference between methods. Overall, SRF is ranked first, followed by RFopt, COBRA, and then RF. Wilcoxon p-values provide strong evidence supporting superiority of SRF to each of the three other methods.

**Table 2:** **Regression benchmark performance**

	**COBRA**	**RF**	**RFopt**	**SRF**
COBRA	**2.7**	0.0968	0.3703	0.0000
RF	388	**3.3**	0.0000	0.0000
RFopt	623	1029	**2.3**	0.0018
SRF	1005	966	827	**1.7**

Upper diagonal values are Wilcoxon signed rank p-values comparing two procedures; lower diagonal values are the corresponding test statistic. Diagonal values (in bold) record the overall rank of a procedure.

### Multiclass results

To further assess the performance of SRF, a total of 38 multiclass benchmark datasets were used. Sample sizes ranged from *n*=29 to *n*=6435; features varied from *p*=2 to *p*=8740; and number of classes *J* varied from *J*=2 to *J*=15 (Table 3). The same nomenclature was adopted as in our regression experiment. Real datasets are indicated with capitals and synthetic data from mlbench are labeled starting with “mlb”. Datasets “aging”, “brain”, “colon”, “leukemia”, “lymphoma” and “srbct” are well-known benchmark microarray datasets (note how *p*≫*n* in each of these).

**Table 3:** **Multiclass benchmark performance**

	***n***	***p***	***J***	**RF**	**RFopt**	**SRF**
BreastCancer	683	10	2	2.59	2.50	2.28
DNA	3186	180	3	3.03	2.79	2.34
Esophagus	3127	28	2	18.33	17.81	18.27
Glass	214	9	6	6.16	6.20	5.78
HouseVotes84	232	16	2	5.85	4.82	4.41
Hypothyroid	2000	24	2	1.20	1.18	1.14
Ionosphere	351	34	2	5.76	5.28	5.14
PimaIndiansDiabetes	768	8	2	15.69	15.66	16.21
Prostate	158	20	2	15.81	15.83	16.02
Satellite	6435	36	6	2.30	1.98	1.92
SickEuthyroid	2000	24	2	2.51	2.35	2.30
Sonar	208	60	2	12.91	12.46	9.73
SouthAfricanHeart	462	9	2	19.69	19.34	19.86
Soybean	562	35	15	0.82	0.71	0.77
Spam	4601	57	2	4.39	4.18	3.74
Vehicle	846	18	4	7.51	8.81	6.82
Vowel	990	10	11	2.66	1.81	1.09
WisconsinBreast	699	10	2	3.13	3.05	3.07
Zoo	101	16	7	1.53	0.51	1.30
aging	29	8740	3	16.64	16.96	16.54
brain	42	5597	5	8.32	7.07	7.99
colon	62	2000	2	12.88	12.78	12.78
leukemia	72	3571	2	4.06	3.95	2.45
lymphoma	62	4026	3	2.71	2.62	2.31
prostate	102	6033	2	8.36	8.25	5.85
srbct	63	2308	4	3.62	3.70	2.45
mlb.cassini	250	2	3	0.92	0.55	0.62
mlb.circle	250	2	2	5.27	4.59	4.26
mlb.cuboids	250	3	4	0.66	0.53	0.57
mlb.dnormals	250	2	2	6.24	6.30	6.31
mlb.ringnorm	250	20	2	10.71	10.17	4.83
mlb.shapes	250	2	4	0.87	0.70	0.52
mlb.smiley	250	2	4	0.51	0.26	0.58
mlb.spirals	250	2	2	1.66	0.72	0.18
mlb.threenorm	250	20	2	15.62	15.34	12.98
mlb.twonorm	250	20	2	8.50	7.87	4.31
mlb.waveform	250	21	3	9.34	9.47	7.83
mlb.xor	250	2	2	3.61	2.50	1.30

Cross-validated and test-set Brier score performance (× 100) over 100 independent replications.

Note first that if a nonparametric regression scheme of any type, learning machine or otherwise, is consistent for the expectation of the outcome, then in a binary or multiclass group membership prediction problem, it necessarily and automatically returns a consistent estimate for the true conditional probability of group membership. Hence, it makes sense to apply a standard measure of probability estimation, so performance was assessed using the classical Brier score (multiplied by 100). The Brier score directly measures accuracy in estimating the true conditional probability, and this is the task of any regression scheme given binary or multiclass group membership. As calibration, we note that a Brier score of 25 represents a procedure with performance no better than random guessing. As in the regression experiment, 10-fold validation was used to estimate performance over real datasets and for synthetic data an independent test-set of size *n*=5000 was used. The entire process was repeated independently 100 times.

**Figure 2:**
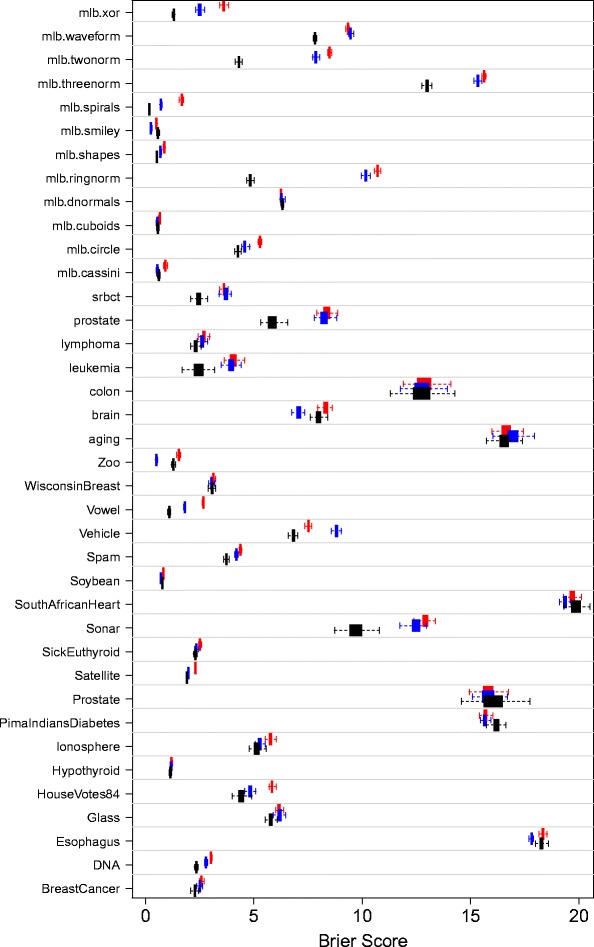
**Multiclass benchmark results.** Cross-validated and test-set Brier score performance (×100) over 100 independent replications. Boxplots display results from the 100 replications for RF (red square symbol), optimized random forests RFopt (blue square symbol), and synthetic random forests SRF (■).

Table 3 and Figure 2 show superiority of SRF to the three other methods. As in the regression experiment, performance differences are especially noticeable over synthetic data. Noticeable performance differences are also observed over certain microarray datasets (srbct, prostate, and leukemia). Table 4 displays results of nonparametric tests comparing procedures. The results parallel those of Table 2: SRF has best overall rank and there is strong evidence of its superiority. The modified Friedman test of equality of procedures yielded a near zero p-value, further confirming evidence of SRF’s superior performance.

**Table 4:** **Multiclass benchmark performance**

	**RF**	**RFopt**	**SRF**
RF	**2.68**	0.0000	0.0000
RFopt	648	**1.86**	0.0045
SRF	688	563	**1.45**

Upper diagonal values are Wilcoxon signed rank p-values comparing two procedures; lower diagonal values are the corresponding test statistic. Diagonal values (in bold) record the overall rank of a procedure.

## Conclusions

Peering more closely at synthetic forests it is possible to discern a reason for the generally good performance of any RF. That is, a single RF is acting as a synthetic machine across all the features, where each original feature is effectively a stand-alone synthetic feature. The manner in which RF synthesizes its features also plays a vital role in its success. RF forms its predictor by taking a locally weighted convex combination of the outcomes. Importantly, this differs from the COBRA method, which locally weights the outcomes using zero-one weights. The superior performance of synthetic forests to COBRA found in our experiments, even when using the same synthetic features as individual, separate constituents in the collective portfolio, suggests that the use of convex, locally determined weights may play a key role in its success, and where these weights are chosen by the refined cells in the data space that are given by the terminal nodes in each tree in each forest.

Performance gains for synthetic forests were most noticeable among the simulated data in our benchmark experiments. We believe the reason for this is that these particular data structures have high signal and sparse solutions. The synthetic random forest, by varying the synthetic inputs over a wide range of user-specified terminal node sizes, acts as a local smoothing optimizer. Our results suggest such tuning is better able to handle high signal, sparse data. Indeed, especially noteworthy given this outcome, is that such data are known to be especially challenging and are likely to constitute a significant fraction of increasingly available big data sets. Another important practical implication of synthetic forests is that the number of RF user tuning parameters are greatly minimized. Most importantly, the synthetic forest does this with evidently no loss in prediction compared to a well-optimized single random forest.

Finally, and more comprehensively, the results here suggest that any statistical learning machine, Super X say, that has user tuning parameters, or indeed required parameter estimation, can be deployed as a Synthetic Super X using RF, with less overhead and likely no real loss in predictive capacity over the fully optimized Super X on the given data.

## Abbreviations

COBRA: COmBined regression alternative
RF: Random forests
RFopt: Nodesize optimized random forests
SRF: Synthetic random forests

## References

[1] Biau G, Fischer A, Guedj B, Malley JD: **COBRA: a non-linear aggregation strategy**. *Paris – France: Technical Report, Université Pierre et Marie Curie;*2013:1–27. [[http://www.lsta.upmc.fr/BIAU/publications.html]]

[2] Vapnik V (1998). Statistical Learning Theory.

[3] Tibshirani RJ (1996). **Regression shrinkage and selection via the lasso**. J R Stat Soc Series B.

[4] Ripley DB (1996). Pattern recognition and neural networks.

[5] Cover TM, Hart PE (1967). **Nearest neighbor pattern classification**. IEEE Trans Inform Theory.

[6] Breiman L: **Random forests**. *Mach Learn*2001, **45:**5.

[7] Pan Q, Hu T, Malley J, Andrew A, Karagas M, Moore J (2014). **A system-level pathway-phenotype association analysis using synthetic feature random forest**. Genet Epidemiol.

[8] Biau G, Devroye L, Lugosi G (2008). **Consistency of random forests and other averaging classifiers**. J Mach Learn Res.

[9] Ishwaran H, Kogalur UB, Chen X, Minn AJ (2011). **Random survival forests for high-dimensional data**. Stat Anal Data Mining.

[10] Lin Y, Jeon Y (2006). **Random forests and adaptive nearest neighbors**. J Am Stat Assoc.

[11] Ishwaran H, Kogalur U: **Random forests for survival, regression and classification (RF-SRC), R package version 1.5.5**2014. [http://cran.r-project.org/web/packages/randomForestSRC/index.html]

[12] Liaw A, Wiener M (2002). **Classification and regression by randomforest**. R News.

[13] Guedj B: **COBRA: nonlinear aggregation of predictors. R package version 0.99.4**2013. [http://cran.r-project.org/web/packages/COBRA/index.html]

[14] Leisch F, Dimitriadou E: **mlbench: machine learning benchmark problems. R package version 2.1-1**2012. [http://cran.r-project.org/web/packages/mlbench/index.html]

[15] Demsar J (2006). **Statistical comparisons of classifiers over multiple data sets**. J Mach Learn Res.

